# Associations Between Physiological Determinants and GNSS-Derived Technical Characteristics in Cross-Country Roller Skiing

**DOI:** 10.3390/s25082521

**Published:** 2025-04-17

**Authors:** Shunya Uda, Naoto Miyamoto, Wako Kajiwara, Hiroshi Nakano, Keisuke Onodera, Ryoji Horimoto, Takato Okada, Masaki Takeda

**Affiliations:** 1 Graduate School of Health and Sports Science, Doshisha University, 1-3 Tatara Miyakodani, Kyotanabe 610-0394, Japan; cyhj0007@mail4.doshisha.ac.jp (S.U.); ctvj0002@mail4.doshisha.ac.jp (W.K.); cyhk0003@mail4.doshisha.ac.jp (H.N.); k-onodera@biwakogakuin.ac.jp (K.O.); ctvk0004@mail4.doshisha.ac.jp (R.H.); ctvk0010@mail4.doshisha.ac.jp (T.O.); 2 Research Center for Sports Sensing, Doshisha University, 1-3 Tatara Miyakodani, Kyotanabe 610-0394, Japan; namiyamo@mail.doshisha.ac.jp; 3 Faculty of Education and Welfare, Biwako-Gakuin University, 29 Fusecho, Higashiomi 527-8533, Japan; 4 Faculty of Health and Sports Science, Doshisha University, 1-3 Tatara Miyakodani, Kyotanabe 610-0394, Japan

**Keywords:** cross-country skiing, physiological determinants, technical characteristics, global navigation satellite system, double poling, G3 skating sub-technique

## Abstract

This study aimed to examine how physiological determinants are associated with skiing technique and race performance in cross-country roller skiing by integrating motion data obtained via a Global Navigation Satellite System (GNSS) with laboratory-based physiological assessments. Nineteen well-trained male skiers completed a 10 km roller ski race, during which skiing velocity, cycle length, cycle time, and sub-technique usage were measured using GNSS. Whole-body and upper-body endurance and power were evaluated on the treadmill and ski ergometer. Time to exhaustion during the double poling test (r = −0.84, *p* < 0.01) and VO_2max_ from the pole walk and run test (r = −0.72, *p* < 0.01) were the strongest predictors of race performance, and both were significantly associated with skiing velocity (VO_2max_: r = 0.79, *p* < 0.01; TTE-DPT: r = 0.81, *p* < 0.01) and cycle length (VO_2max_: r = 0.58, *p* < 0.01; TTE-DPT: r = 0.47, *p* < 0.05) in the most frequently used technique. These findings suggest that the development of both whole-body and upper-body endurance plays a crucial role in improving technical efficiency and race performances.

## 1. Introduction

The association between cross-country skiing performance and physiological determinants has been extensively investigated through field and laboratory tests. Among these determinants, relative maximal oxygen uptake per body weight (VO_2max_/kg) is widely recognized as a key indicator of whole-body endurance and is strongly associated with performance [1,2,3]. Additionally, recent studies have highlighted the importance of double poling (DP) ability in cross-country skiing, emphasizing its critical role in enhancing competitive performance [4,5,6,7,8]. For instance, sport-specific roller skiing tests have demonstrated that relative peak oxygen uptake per body weight (VO_2peak_/kg) achieved during DP is significantly higher in skiers with superior performance levels [5]. Furthermore, upper-body power measured on a ski ergometer has also shown strong correlations with performance outcomes [6]. These findings suggest that sport-specific physiological determinants, particularly those associated with DP, are crucial determinants of cross-country skiing performance.

In addition to physiological determinants, performance improvements in cross-country skiing have been attributed to increased usage rates of faster sub-techniques [9,10] and extended cycle length [11]. Equipment advancements, including skis, poles, and boots, and improvements in ski track precision, waxing, and structuring techniques, are believed to have significantly contributed to increased skiing velocity. Alongside these external improvements, skiers’ improved physical fitness is also likely to play a pivotal role. However, the mechanisms by which these external improvements are supported or influenced by internal metabolic and neuromuscular adaptations still need to be discovered. For example, the close association between upper-body power and technical characteristics [7] suggests an interactive evolution of physiological attributes and technical strategies, yet the underlying mechanisms remain unclear.

Recent advancements in sensing technologies, such as the Global Navigation Satellite System (GNSS) and inertial measurement units (IMUs), have enabled the high-precision kinematic and kinetic data collection under race conditions [12,13,14]. These technological advancements provide a foundation for further exploring the association between physiological characteristics and technical strategies. Nonetheless, much of the current research remains correlational, providing limited insight into the causal mechanisms through which physiological adaptations and technical innovations interact to enhance performance.

From an integrated perspective, this study aimed to comprehensively reexamine physiological determinants, including whole-body endurance, upper-body endurance, and upper-body power. It further proposes a novel framework to elucidate the causal mechanisms by which these determinants contribute to the selection, maintenance, and optimization of sub-techniques. This study hypothesizes those physiological determinants of cross-country skiing influence not only overall performance but also the selection and optimization of sub-techniques. These determinants are expected to correlate with key kinematic variables such as skiing velocity, cycle length, and cycle time, under actual race conditions. The outcomes of this study are expected to provide practical insights for coaching and training program development while also fostering new research directions and methodological advancements. This work aims to provide new insights into the interaction between physiology and technique, contributing to the advancement of cross-country skiing research.

## 2. Materials and Methods

### 2.1. Participants

Nineteen well-trained male cross-country skiers volunteered as subjects. (age: 19.9 ± 3.1 years; height: 170.5 ± 5.6 cm; weight: 64.7 ± 7.8 kg; VO_2max_/kg: 69.6 ± 4.9 mL·kg^−1^·min^−1^). Their performance levels ranged from high school students to national-level skiers. The study was approved by the Doshisha University Research Ethics Committee and conducted in accordance with the Declaration of Helsinki (No. 23042). All participants provided written informed consent after explaining the study’s purpose and procedures.

### 2.2. Overall Design

The subjects participated in a 10 km roller ski skating timed race (3 laps of 3.5 km) held in early September. During the race, each subject was equipped with a high-precision GNSS device to record race time and technical characteristics, including sub-technique usage ratio by time, skiing velocity, cycle length, and cycle time. In October, a series of laboratory tests were conducted, including the pole walk and run test (PWT), the double pole test (DPT), and the ski ergometer test (SET). All participants completed these tests within a three-week period. During the interval between the timed race and laboratory tests, the subjects followed their usual training routines, and there were no reports of injuries or illnesses that might have significantly affected their physical condition.

### 2.3. Timed Race

**Figure 1:**
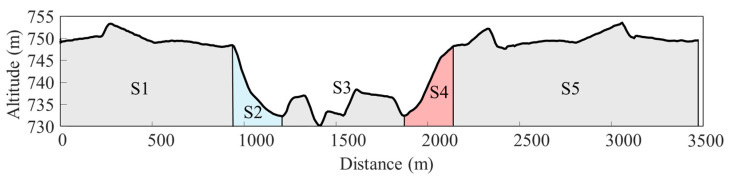
Course profile of the timed race. The course used for the race was the Kijimadaira Cross-Country Ski Course (Course No. SAJ06-CC-56/99) located in Nagano, Japan.

The subjects performed a self-selected warm-up for up to 40 min prior to the timed race. A GNSS antenna (lightweight helical GNSS triple band + L-band antenna, ArduSimple, Lleida, Spain) was secured to the subject’s helmet, while the receiver (AT-H-35 rev.B, AOBA Technologia, Sendai, Japan) and Wi-Fi router (Aterm MP01LN, NEC, Tokyo, Japan) were placed in the vest pocket [15]. The subjects used their own poles and shoes, while standardized roller skis (MS610A Skating model, Marwe, Hyvinkää, Finland) and wheels (Skate Wheel US0, Marwe, Hyvinkää, Finland) were provided to ensure consistency. In addition, they wore a heart rate monitor (Polar Vantage V2, Polar Electro Oy, Kempele, Finland) and a heart rate sensor (Polar H10N, Polar Electro Oy, Kempele, Finland) to measure heart rate during the timed race. A 20 min warm-up using the roller skis was conducted to stabilize the wheel performance. Weather conditions were favorable, with clear skies, 28–32 °C temperatures, 55–70% humidity, and minimal wind. The subjects started individually at 15 s intervals. Race time was recorded using the GNSS device, capturing the moment each subject’s head crossed the start and finish lines. Terrain sections were classified based on the International Ski Federation (FIS) Homologation Manual for cross-country skiing courses [16]. Gradient changes in the course profile were used to define section boundaries. The uphill section (S4) was defined as having a climb of more than 10 m with a gradient of 5% or greater, while the downhill section (S2) involved a descent of more than 10 m with a gradient of −5% or steeper. Intermediate sections (S1, S3, S5) included short climbs, descents, or flat areas (Figure 1). All subjects were instructed to limit their training load to low-intensity sessions lasting no more than 1.5 h during the two days prior to the race. They were also advised to avoid consuming large meals within two hours of the race.

### 2.4. Sub−Technique Classification and Technical Characteristics

There are seven sub-techniques (G1–G7) in the skating style. G1 involves pushing with one ski while simultaneously using the opposite pole and is primarily employed on very steep uphill sections or when the skier is fatigued. G2 is used on uphill sections and involves one asymmetrical poling action for every two leg movements. G3 is applied on flat to gradual uphill sections and features one poling action and one leg movement. G4 is used on flat sections and involves one symmetrical poling action for every two leg movements. G5 relies primarily on the skier’s legs for propulsion without poling action; it is typically employed on flat or slightly descending terrain. G6 is a curve-specific technique combining leg strokes with or without poling action. Finally, G7 is utilized during downhill skiing, where the skier adopts a tuck position without poling action or leg movements [9,17,18]. In this study, sub-techniques were classified as G2, G3, G4, G6, and downhill. G1 was not used in any of the subjects. G6 refers exclusively to turns with poling action, while G6 refers to turns without poling action; G5 and G7 were collectively classified as downhill techniques (DH). Movements involving DP, loss of balance, or poor GNSS signal reception that hindered accurate classification were classified as “others”. The technical characteristics of each sub-technique were visually analyzed based on the waveform data of vertical and lateral head movements, as well as ground speed obtained from the GNSS device mounted on the subjects’ heads [14]. This method, adapted to the experimental conditions on varied paved road courses, was validated using video recordings, achieving a high classification accuracy of 98.5%. The technical characteristics of each sub-technique, including usage time ratio, skiing velocity, cycle length, and cycle time, were analyzed for the entire race. In addition, specific analyses were conducted for G3 in the intermediate (S1, S3, S5) and uphill (S4) sections.

### 2.5. Laboratory Tests

The laboratory tests were conducted over two consecutive days. On the first day, the PWT was performed, followed by the DPT and SET on the second day. These tests were familiar to the subjects as they were regularly incorporated into their training routines, ensuring that the subjects were well-practiced and capable of performing at their best during the assessments. To minimize the effects of fatigue, a two-hour recovery period was scheduled between the DPT and SET to allow adequate rest. Tests were conducted in a controlled environment with a temperature of 21–22 °C, humidity of 40–60%, and continuous ventilation. Before each test, the subjects completed a self-selected warm-up to ensure safety and maintain optimal physical condition. Additionally, they were instructed to limit training intensity to low-intensity sessions lasting more than 1.5 h during the two days preceding the tests and to avoid consuming large meals within two hours of the tests. During the recovery period between the DPT and SET, the subjects were allowed to consume light, energy-dense foods and drinks to support recovery and maintain performance.

The PWT was conducted to assess whole-body endurance and physiological responses during exercise. The test was performed on a treadmill measuring 1.0 m in width and 2.7 m in length (RL 2700RL, Rodby, Uppsala, Sweden), equipped with a slip-resistant rubber belt for safety and reliability. The subjects wore their own shoes and poles, fitted with carbide tips for optimal grip.

The progressive load protocol began at 6 km/h with an incline of 6 degrees. During the first 15 min, speed and incline increased every two minutes. Thereafter, speed alone increased by 0.6 km/h every minute (Table 1). The subjects began by walking with poles and transitioned to running as the workload intensified until exhaustion.

Respiratory variables were measured using a pulmonary exercise monitoring system (Aero Monitor AE-310S, Minato Medical Science Co., Ltd., Osaka, Japan). This system employs a breath-by-breath measurement method using a hot-wire flowmeter for real-time analysis of respiratory flow and gas concentrations, calculating respiratory metabolic indices. The system was calibrated before each session using ambient air conditions and certified reference gases (O_2_ 15.0%; CO_2_ 5.06%). A precision calibration syringe (2 L, Hans Rudolph, Shawnee, KS, USA) was used to calibrate the flow transducer. Heart rate was recorded using a heart rate monitor (Polar H10 N, Polar Electro Oy, Kempele, Finland), synchronized with the pulmonary exercise monitoring system.

Primary analyses focused on the time to exhaustion in the PWT (TTE-PWT), absolute maximal oxygen uptake (VO_2max_), VO_2max_/kg, fractional utilization of VO_2max_ (%VO_2max_/kg) at the ventilatory threshold (VT), and %VO_2max_/kg at the respiratory compensation threshold (RCT). VT and RCT were determined following the methodology described by Binder et al. [19]. VT was identified using the V-slope method, which analyzes the VCO_2_ vs. VO_2_ double regression line to detect the point where the slope transitions from less than one to greater than or equal to one. VT was also determined as the minimum point or the first increase in VE/VO_2_ against workload, provided there was no simultaneous increase in VE/VCO_2_. Additionally, VT was identified as the minimum point or the first increase in PETO_2_ (end-tidal oxygen partial pressure) with stable or increasing PETCO_2_ (end-tidal carbon dioxide partial pressure). RCT was determined based on the inflection point in VE vs. VCO_2_, the minimum point or a nonlinear increase in VE/VCO_2_ against workload, and the inflection point in PETCO_2_. These determinations were independently confirmed by two observers through visual inspection to ensure reliability. Heart rate data were excluded from the analysis. For VO_2max_ and threshold determinations, breath-by-breath data were collected at 15 s intervals, converted to minute-by-minute averages, and the highest averaged value was adopted as VO_2max_. Similarly, threshold points were determined using per-minute average data and identified according to Binder’s definitions.

The DPT was conducted to evaluate upper-body endurance during DP, a key technique in cross-country skiing. All subjects used identical skating roller skis (MC700XC Classic, MARWE, Hyvinkää, Finland) with identical wheels (Classic Wheel SC0, MARWE, Hyvinkää, Finland). They wore their own boots and poles, fitted with carbide tips for optimal grip. A ceiling-suspended safety harness ensured continuous safety during the test. A 20 min warm-up on the roller skis was performed to stabilize wheel performance before commencing the test. The test protocol began at an initial 10 km/h speed with a 2.0-degree incline. The incline increased by 1.0 degrees every minute to progressively intensify the workload (Table 1). The treadmill, respiratory measurement system, and heart rate monitor used during the experiment were identical to those employed in the PWT, ensuring consistent measurement conditions. During the test, time to exhaustion in the DPT (TTE-DPT), absolute peak oxygen uptake (VO_2peak_), and VO_2peak_/kg were recorded. VO_2peak_ was determined using the same manner as VO_2max_ in the PWT—breath-by-breath data were collected at 15 s intervals, converted to minute-by-minute averages, and the highest averaged value was adopted as VO_2peak_. Additionally, the ratio of VO_2peak_ in the DPT to VO_2max_ in the PWT was calculated to assess the VO_2peak_ to VO_2max_ ratio (%VO_2peak_/VO_2max_) specific to DP. Heart rate data were excluded from the analysis.

The SET assessed the subjects’ upper-body power during a controlled DP. The test used a Nordic ski ergometer (Concept2 Ski Ergometer, Concept2., Morrisville, VT, USA) equipped with ski bindings, and the subjects wore ski boots with their toes securely fixed to ensure stability. The subjects performed DP for 60 s with instructions to achieve the highest possible mean power output. The air brake of the ergometer was set to maximum resistance throughout the test. Key outcomes included mean power and relative mean power per body weight (mean power/kg), providing a standardized assessment of the subjects’ physical capacity.

### 2.6. Data Analysis

All data were tested for normality using the Shapiro–Wilk test and are presented as mean ± standard deviation (SD). The analysis included race time, time usage ratio, skiing velocity, cycle length, and cycle time for the entire race and a specific section (S4), as well as the results of laboratory tests (PWT, DPT, and SET). Time usage ratio, skiing velocity, cycle length, and cycle time for each sub-technique during the race were analyzed using analysis of variance (ANOVA). When necessary, Bonferroni post hoc tests were applied to identify pairwise differences. Additionally, Pearson correlation analysis was performed to examine the associations among 10 km skating race time, technical characteristics of each sub-technique, and physiological parameters obtained from laboratory tests. Correlation strength was interpreted using the criteria proposed by Hopkins et al. [20]: trivial (r < 0.1), small (0.1 ≤ r < 0.3), moderate (0.3 ≤ r < 0.5), large (0.5 ≤ r < 0.7), very large (0.7 ≤ r < 0.9), and extremely large (r ≥ 0.9). Stepwise multiple regression analysis was conducted to identify predictors of roller ski race time, with race time set as the dependent variable and laboratory test results as explanatory variables. Only variables showing significant correlations with race time were included in the regression model. A significant level of *p* < 0.05 was applied for all statistical tests. All statistical analyses were performed using MATLAB (R2024a, The MathWorks, Natick, MA, USA), with the Statistics and Machine Learning Toolbox for advanced data analysis.

## 3. Results

### 3.1. The Association Between Race Time and Physiological Parameters

The mean race time was 1539.7 ± 153.2 s. The average maximum heart rate during the timed race was 189.7 ± 10.5 bpm. Table 2 presents the descriptive statistics for each test and the correlation coefficients between race time and physiological Parameters. For the PWT, the mean TTE-PWT was 1046.5 ± 81.6 s, VO_2max_ was 4455.9 ± 517.1 mL·min^−1^, VO_2max_/kg was 69.6 ± 4.9 mL·kg^−1^·min^−1^, % VO_2max_/kg at VT was 58.3 ± 7.2%, and % VO_2max_/kg at RCT was 78.2 ± 5.3%. For the DPT, the mean TTE-DPT was 371.9 ± 55.6 s, VO_2peak_ was 3761.6 ± 554.2 mL·min^−1^, VO_2peak_/kg was 58.8 ± 5.4 mL·kg^−1^·min^−1^, and %VO_2peak_/VO_2max_ was 84.4 ± 6.0%. For the SET, the mean power was 369.3 ± 63.8 W, and the mean power/kg was 5.7 ± 0.9 W·kg^−1^. Correlation analysis revealed very large negative correlations between race time and TTE-DPT (r = −0.84, *p* < 0.01), TTE-PWT (r = −0.75, *p* < 0.01), VO_2peak_ (r = −0.76, *p* < 0.01), and VO_2max_ (r = −0.72, *p* < 0.01). Large negative correlations were observed for %VO_2max_/kg at VT (r = −0.56, *p* < 0.05), %VO_2max_/kg at RCT (r = −0.51, *p* < 0.05), VO_2peak_/kg (r = −0.59, *p* < 0.01), and mean power in the SET (r = −0.53, *p* < 0.05). In contrast, no significant correlations were found for %VO_2peak_/VO_2max_ (r = −0.39, *p* = n.s.; moderate), VO_2max_/kg (r = −0.32, *p* = n.s.; moderate), and mean power/kg (r = −0.26, *p* = n.s.; small).

Stepwise regression analysis showed that TTE-DPT was the first variable to enter the model, followed by VO_2max_, and, finally, the interaction term VO_2max_: TTE-DPT. The final regression model was expressed as follows:

Race Time = 4634.6 − 0.54793·VO_2max_ − 7.0353·TTE-DPT + 0.0011732·(VO_2max_ × TTE-DPT)

The estimated coefficients were as follows: Intercept = 4634.6 (*p* < 0.01), VO_2max_ = −0.54793 (*p* < 0.01), TTE-DPT = −7.0353 (*p* < 0.01), and VO_2max_: TTE-DPT = 0.0011732 (*p* < 0.01). Based on 19 observations (with 15 degrees of freedom for error), the model yielded a root mean square error (RMSE) of 62.2, an R^2^ of 0.86, and an adjusted R^2^ of 0.83. Compared with the intercept-only model, this final model demonstrated a strong fit for the data. (F = 31.4, *p* < 0.01). The cross-validation mean squared error (MSE) was 3054.9, and the standard error of the estimate (SEE) was 62.2, indicating strong predictive performance.

### 3.2. Technical Characteristics During the Roller Ski Timed Race

**Figure 2:**
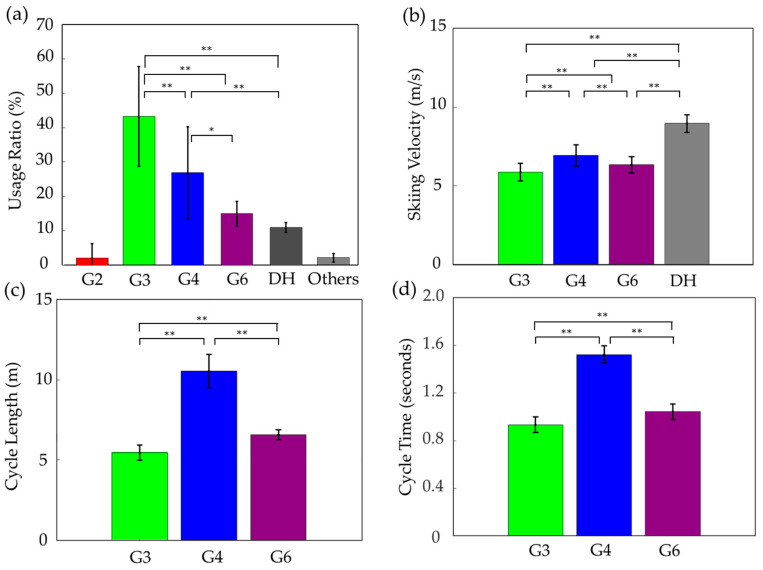
Technical characteristics during the roller ski timed race, including (**a**) time-based usage ratio, (**b**) skiing velocity, (**c**) cycle length, and (**d**) cycle time. ** *p* < 0.01, * *p* < 0.05.

The mean time-based usage ratios for sub-techniques during the roller ski race were as follows (Figure 2a). For G2, the ratio was 1.99 ± 4.29%, for G3, 43.15 ± 14.58%, for G4, 26.86 ± 13.41%, for G6, 14.99 ± 3.62%, for DH, 10.42 ± 2.67%, and for Others, 2.58 ± 2.29%. G2 was primarily used at the start of the race, and only four participants used it outside of the start phase. Therefore, it was excluded from further analysis. The mean skiing velocities were as follows (Figure 2b). For G3, the mean velocity was 5.89 ± 0.56 m/s, for G4, 6.95 ± 0.67 m/s, for G6, 6.34 ± 0.51 m/s, and for DH, 8.97 ± 0.55 m/s. The mean cycle lengths for G3, G4, and G6 were as follows (Figure 2c). For G3, the mean cycle length was 5.46 ± 0.47 m, for G4, 10.54 ± 1.03 m, and for G6, 6.58 ± 0.30 m. The mean cycle times for G3, G4, and G6 were as follows (Figure 2d). For G3, the mean cycle time was 0.93 ± 0.06 s, for G4, 1.52 ± 0.07 s, and for G6, 1.04 ± 0.06 s.

### 3.3. The Associations Between Technical Characteristics and Physiological Parameters

The correlations between the time usage ratio, skiing velocity, cycle length, and cycle time with race time and physiological parameters were analyzed for each sub-technique, including G3, G4, G6, and DH, during the timed race. The results are as follows (Table 3). For G3, skiing velocity exhibited a very large negative correlation with race time (r = −0.89, *p* < 0.01) and very large positive correlations with TTE-PWT (r = 0.70, *p* < 0.01), VO_2max_ (r = 0.79, *p* < 0.01), TTE-DPT (r = 0.81, *p* < 0.01), and VO_2peak_ (r = 0.81, *p* < 0.01). It also showed large positive correlations with %VO_2max_/kg at VT (r = 0.59, *p* < 0.01), VO_2peak_/kg (r = 0.56, *p* < 0.05), and mean power (r = 0.51, *p* < 0.05). Cycle length displayed large negative and large positive correlations with race time (r = −0.53, *p* < 0.05) and VO_2max_ (r = 0.58, *p* < 0.01), respectively, as well as moderate to large associations with %VO_2max_/kg at VT (r = 0.54, *p* < 0.05), TTE-DPT (r = 0.47, *p* < 0.05), and VO_2peak_ (r = 0.58, *p* < 0.01). In contrast, cycle time demonstrated large positive correlations with race time (r = 0.62, *p* < 0.01) and negative correlations with TTE-PWT (r = −0.64, *p* < 0.01), VO_2max_/kg (r = −0.54, *p* < 0.05), TTE-DPT (r = −0.55, *p* < 0.05), and VO_2peak_/kg (r = −0.62, *p* < 0.01). For G4, skiing velocity revealed a very large negative correlation with race time (r = −0.88, *p* < 0.01) and very large positive correlations with TTE-PWT (r = 0.76, *p* < 0.01), VO_2max_ (r = 0.75, *p* < 0.01), TTE-DPT (r = 0.81, *p* < 0.01), and VO_2peak_ (r = 0.82, *p* < 0.01). It also showed large positive to moderate positive correlations with VO_2peak_/kg (r = 0.61, *p* < 0.01), %VO_2max_/kg at VT (r = 0.51, *p* < 0.05), %VO_2max_/kg at RCT (r = 0.48, *p* < 0.05), and mean power (r = 0.47, *p* < 0.05). Cycle length had a very large negative correlation with race time (r = −0.75, *p* < 0.01) but correlated positively in the large range with TTE-PWT (r = 0.59, *p* < 0.01) and VO_2max_ (r = 0.58, *p* < 0.01), respectively, as well as with TTE-DPT (r = 0.64, *p* < 0.01) and VO_2peak_ (r = 0.68, *p* < 0.01). A moderate positive correlation was observed with %VO_2max_/kg at VT (r = 0.48, *p* < 0.05). Cycle time was moderately negatively correlated with VO_2max_/kg (r = −0.48, *p* < 0.05). For G6, the time-based usage ratio showed a large negative correlation with race time (r = −0.62, *p* < 0.01) and large positive correlations with TTE-DPT (r = 0.50, *p* < 0.05), VO_2peak_/kg (r = 0.52, *p* < 0.05), mean power (r = 0.54, *p* < 0.05), and mean power/kg (r = 0.54, *p* < 0.05). Its correlation with VO_2peak_ (r = 0.46, *p* < 0.05) was moderately positive. Skiing velocity exhibited a nearly perfect negative correlation with race time (r = −0.97, *p* < 0.01) and very large positive correlations with TTE-PWT (r = 0.78, *p* < 0.01), VO_2max_ (r = 0.73, *p* < 0.01), TTE-DPT (r = 0.85, *p* < 0.01), and VO_2peak_ (r = 0.78, *p* < 0.01). It also showed large positive correlations with %VO_2max_/kg at VT (r = 0.57, *p* < 0.05), %VO_2max_/kg at RCT (r = 0.50, *p* < 0.05), VO_2peak_/kg (r = 0.59, *p* < 0.01), and mean power (r = 0.53, *p* < 0.05). Cycle length demonstrated a very large negative correlation with race time (r = −0.70, *p* < 0.01) and large positive correlations with VO_2max_ (r = 0.53, *p* < 0.05), TTE-DPT (r = 0.54, *p* < 0.05), and VO_2peak_ (r = 0.55, *p* < 0.05). Conversely, cycle time showed a very large positive correlation with race time (r = 0.83, *p* < 0.01) and very large negative correlations with TTE-PWT (r = −0.77, *p* < 0.01) and TTE-DPT (r = −0.76, *p* < 0.01). It also had large negative correlations with VO_2max_ (r = −0.61, *p* < 0.01), %VO_2max_/kg at RCT (r = −0.55, *p* < 0.05), VO_2peak_ (r = −0.66, *p* < 0.01), and VO_2peak_/kg (r = −0.66, *p* < 0.01). For DH, skiing velocity displayed a nearly perfect negative correlation with race time (r = −0.92, *p* < 0.01) and very large positive correlations with TTE-PWT (r = 0.79, *p* < 0.01), VO_2max_ (r = 0.76, *p* < 0.01), TTE-DPT (r = 0.85, *p* < 0.01), and VO_2peak_ (r = 0.81, *p* < 0.01). It also showed a large positive correlation with VO_2peak_/kg (r = 0.67, *p* < 0.01) and mean power (r = 0.59, *p* < 0.01).

### 3.4. The Association Between G3 Technical Characteristics and Physiological Parameters Across Terrain

The mean and SD of skiing velocity, cycle length, and cycle time on intermediate and uphill sections were analyzed: The skiing velocity was 6.29 ± 0.56 m/s on intermediate sections and 4.68 ± 0.44 m/s on uphill sections. The cycle length was 5.92 ± 0.50 m on intermediate sections and 4.18 ± 0.31 m on uphill sections. The cycle time was 0.94 ± 0.06 s on intermediate sections and 0.90 ± 0.06 s on uphill sections. Significant differences were observed in all parameters between intermediate and uphill sections (all *p* < 0.01). The results further revealed several significant correlations (Table 4). For the PWT, skiing velocity was positively correlated with TTE-PWT (Intermediate: r = 0.72, *p* < 0.01; Uphill: r = 0.68, *p* < 0.01), VO_2max_ (Intermediate: r = 0.76, *p* < 0.01; Uphill: r = 0.67, *p* < 0.01), %VO_2max_/kg at VT (Intermediate: r = 0.62, *p* < 0.01; Uphill: r = 0.59, *p* < 0.01). The cycle length showed significant positive correlations with VO_2max_ (Intermediate: r = 0.55, *p* < 0.05; Uphill: r = 0.49, *p* < 0.05) and %VO_2max_/kg at VT (Intermediate: r = 0.54, *p* < 0.05; Uphill: r = 0.58, *p* < 0.01). Conversely, cycle time was negatively correlated with TTE-PWT (Intermediate: r = −0.61, *p* < 0.01; Uphill: r = −0.62, *p* < 0.01), VO_2max_/kg (Intermediate: r = −0.53, *p* < 0.05; Uphill: r = −0.54, *p* < 0.05). For DPT, skiing velocity was positively correlated with TTE-DPT (Intermediate: r = 0.83, *p* < 0.01; Uphill: r = 0.81, *p* < 0.01), VO_2peak_ (Intermediate: r = 0.79, *p* < 0.01; Uphill: r = 0.72, *p* < 0.01), and VO_2peak_/kg (Intermediate: r = 0.56, *p* < 0.05; Uphill: r = 0.62, *p* < 0.01). The cycle length showed significant positive correlations with TTE-DPT (Uphill: r = 0.58, *p* < 0.01), VO_2peak_ (Intermediate: r = 0.53, *p* < 0.05; Uphill: r = 0.54, *p* < 0.05). Cycle time was negatively correlated with TTE-DPT (Intermediate: r = −0.56, *p* < 0.05; Uphill: r = −0.53, *p* < 0.05) and VO_2peak_/kg (Intermediate: r = −0.64, *p* < 0.01; Uphill: r = −0.58, *p* < 0.01). For SET, skiing velocity showed significant positive correlations with mean power (Intermediate: r = 0.52, *p* < 0.05; Uphill: r = 0.55, *p* < 0.05). Additionally, cycle length was positively correlated with mean power on uphill sections (r = 0.54, *p* < 0.05). No significant correlations were observed between cycle time and physiological parameters of SET.

## 4. Discussion

This study investigated the influence of physiological determinants on race time and technical characteristics in cross-country roller skiing. The results indicated that VO_2max_ and TTE-DPT, measured as physiological parameters, were the primary determinants of race time. VO_2max_, a traditional determinant of whole-body endurance, exhibited a strong negative correlation with race time, reaffirming its importance. TTE-DPT, reflecting upper-body endurance, had the most substantial impact on race time, emphasizing the critical role of DP—a key movement in cross-country skiing—in competitive performance. Furthermore, the interaction between VO_2max_ and TTE-DPT significantly influenced race time, suggesting a synergistic mechanism in which whole-body endurance and upper-body endurance complement each other to optimize performance. These insights underscore the necessity of enhancing whole-body endurance and upper-body endurance in training strategies for cross-country skiing.

Although VO_2max_/kg has traditionally been considered a key physiological determinant of performance, this study found no significant correlation between VO_2max_/kg and race time. Previous studies [21,22,23] have similarly reported a lack of significant associations between conventional physiological parameters and performance, likely due to differences in participant group characteristics or course conditions. In this study, the relatively gentle course profile may have reduced the impact of body weight on performance, explaining the lack of a significant correlation between VO_2max_/kg and race time. Lower body weight is generally advantageous on steep climbs. However, this effect may have been minimized in this study due to the course profile. By contrast, the significant correlation observed between VO_2peak_/kg and race time suggests that upper-body movement-specific abilities, such as DP, may have played a more substantial role in performance.

Additionally, no significant correlation was observed between %VO_2peak_/VO_2max_ and race time. One possible explanation is that the absolute value of VO_2peak_/kg may directly reflect performance characteristics. Moreover, as %VO_2peak_/VO_2max_ is a ratio, a strong correlation between its numerator (VO_2peak_) and denominator (VO_2max_) may have reduced inter-individual variability, making it more challenging to detect statistical significance.

The analysis of technical characteristics revealed distinct patterns in skiing velocity, cycle length, and cycle time influenced by physiological parameters. Among these, the G3 technique was the most frequently used technique during the race, indicating its significant contribution to performance. High-speed techniques such as G3 and G4 exhibited strong positive correlations with VO_2max_ and %VO_2max_/kg at VT (whole-body endurance), as well as TTE-DPT and VO_2peak_ (upper-body endurance), particularly regarding skiing velocity and cycle length. These high-speed techniques were most effective on terrain with lower resistance, such as intermediate sections and gentle uphill slopes. Subjects with higher VO_2max_, %VO_2max_/kg at VT, TTE-DPT, and VO_2peak_ could sustain greater speeds and longer cycle lengths. Since cycle length is a primary determinant of skiing velocity and is closely linked to skiing efficiency and performance level [24]. Our findings indicate that whole-body endurance and upper-body endurance may collectively extend cycle length and enhance skiing velocity. Furthermore, the cycle time analysis showed significant correlations between the cycle times of G3 and G6 and race performance. Higher cycle rates are associated with maximum velocity capabilities, and self-selected cycle rates have been reported to improve efficiency at submaximal velocities [24]. Although VO_2max_/kg did not directly correlate with race time, a significant association between cycle time and race time was observed. Additionally, the cycle time of G3 and G4 was correlated with VO_2max_/kg, suggesting that these physiological parameters may indirectly influence performance. A notable finding was the negative correlation between race time and the time-based usage ratio of G6. While G6 is often viewed as a technique primarily used for directional changes, our results indicate that its effective use can enhance speed rather than simply correct trajectory. Specifically, G6 appears to incorporate DP during directional changes, as evidenced by its positive correlations with TTE-DPT and SET performance. Although G6 accounted for only around 15% of total technique usage, it played a strategically important role in enabling skiers to maintain or increase velocity on undulating terrain.

Finally, analysis of technical characteristics across different terrains revealed that, on uphill sections, cycle time and length decreased simultaneously, leading to reduced skiing velocity. Although no significant differences in physiological parameters were detected between terrains, TTE-DPT and mean power measured during the SET showed significant correlations with technical characteristics on uphill sections. These findings suggest that upper-body power and whole-body endurance, particularly in DP movements, are crucial for maintaining cycle length and improving skiing efficiency when skiing uphill. These insights clarify how physiological parameters influence technique selection and terrain adaptability, shedding light on the role of specific physiological indices in shaping technical efficiency and overall race performance. The findings align closely with the conceptual framework proposed by Losnegard [25], emphasizing the interplay between aerobic energy systems, upper-body endurance, technique selection, and pacing strategies in cross-country roller skiing performance. This study empirically demonstrated strong associations between absolute VO_2max_ and upper-body endurance (TTE-DPT) with sub-technique usage (e.g., G3, G4), skiing velocity, cycle length, and cycle time, thereby reinforcing the importance of both whole-body-specific and upper-body-specific capacities highlighted by Losnegard [25].

The significance of this study lies in its demonstration of the specific impacts of laboratory−measured physiological determinants on race performance and technical characteristics. Utilizing GNSS devices enabled high-precision analysis of the associations between technical characteristics and physiological determinants under actual race conditions, representing a groundbreaking advancement. This approach revealed direct associations between skiers’ technical characteristics and physiological determinants, which traditional laboratory measurements or single-method data collection cannot capture. The findings provide practical insights for designing training programs and strategies, particularly optimizing technique selection and terrain adaptability based on individual skiers’ physiological characteristics.

However, this study has certain limitations. The small sample size necessitates cautious interpretation of the results. Additionally, the diverse age range and competitive levels of the subjects indicate the need for future analysis within a more homogeneous group of high-level skiers. Moreover, as the study was conducted on a course with gentle inclines, the results may not fully apply to steeper courses where techniques such as G2 are more frequently used. Future studies should explore steeper courses to assess their impact on skiing techniques and performance. Since this study was not conducted on snow, further investigation of snow conditions is necessary. The methodology used in this study is also applicable to other endurance sports beyond cross-country skiing. Integrating GNSS technology with movement characteristic data offers a novel approach to enhancing performance in various sports. This broader applicability underscores the practical and scientific contributions of this study. The findings strengthen the scientific foundation of cross-country skiing by providing quantitative evidence of how physiological determinants influence technique selection and technical characteristics. These insights present specific training guidelines to improve athletic performance and contribute meaningfully to the advancement of competitive sports science.

## 5. Conclusions

This study comprehensively examined the potential influence of key physiological determinants on technical characteristics and performance in cross-country roller skiing. A 10 km roller skiing timed race was conducted using high-precision GNSS devices for field data collection and laboratory assessments, including the PWT, DPT, and SET. These evaluations yielded the following key findings:

The results of this study suggest that performance is associated not only with whole-body endurance but also with upper-body power and endurance. Various physiological determinants intricately influence technical characteristics such as skiing velocity, cycle length, and cycle time. Improvements in these physiological capacities collectively contribute to enhanced race performance, emphasizing the interconnected nature of endurance. These results help athletes and coaches identify the physiological indicators necessary to develop higher-performing techniques, thereby enabling evidence-based training design. In particular, a high whole-body VO_2max_ and VO_2peak_ during double poling were strongly associated with the cycle length of the G3 and G4 techniques and emerged as key performance determinants. These findings highlight the importance of training aimed at enhancing both whole-body endurance and double poling capacity.

## Figures and Tables

**Table 1:** Protocol for PWT and DPT.

Protocol for Pole Walk and Run Test (PWT)			Protocol for Double Poling Test (DPT)		
Time (min)	Inclination (degrees)	Speed (km/h)	Time (min)	Inclination (degrees)	Speed (km/h)
0–2	4.2	6.0	0–1	2.0	10.0
2–4	5.3	6.5	1–2	3.0	10.0
4–6	6.3	7.0	2–3	4.0	10.0
6–8	7.9	7.0	3–4	5.0	10.0
8–10	9.5	7.0	4–5	6.0	10.0
10–12	11.1	7.0	5–6	7.0	10.0
12–14	12.7	7.0	6–7	8.0	10.0
14–15	14.0	7.6	7–8	9.0	10.0
15–16	14.0	8.2	8–9	10.0	10.0
From 15 min onward, the incline remains the same, and the speed increases by 0.6 km/h every minute.			The incline increases by 1 degree every minute, and the speed remains constant.		

**Table 2:** The association between race time and physiological parameters. ** *p* < 0.01, * *p* < 0.05.

		Mean ± SD (n = 19)	Correlation with Race Time
Timed Race	Race Time (s)	1539.7 ± 153.2	
PWT	TTE (s)	1046.5 ± 81.6	−0.75 **
	VO_2max_ (mL·min^−1^)	4455.9 ± 517.1	−0.72 **
	VO_2max_/kg (mL·kg^−1^·min^−1^)	69.6 ± 4.9	−0.32
	%VO_2max_ at VT (%)	58.3 ± 7.2	−0.56 *
	%VO_2max_ at RCT (%)	78.2 ± 5.3	−0.51 *
DPT	TTE (s)	371.9 ± 55.6	−0.84 **
	VO_2peak_ (mL·min^−1^)	3761.6 ± 549.2	−0.76 **
	VO_2peak_/kg (mL·kg^−1^·min^−1^)	58.8 ± 5.4	−0.59 **
	%VO_2peak_/VO_2max_ (%)	84.4 ± 6.0	−0.39
SET	Mean power (W)	369.3 ± 63.8	−0.53 *
	Mean power/kg (W·kg^−1^)	5.7 ± 0.9	−0.26

**Table 3:** The associations between technical characteristics and physiological parameters during the roller ski timed race. ** *p* < 0.01, * *p* < 0.05.

		Time-Based Usage Ratios (%)				Skiing Velocity (m/s)				Cycle Length (m)			Cycle Time (s)		
		G3	G4	G6	DH	G3	G4	G6	DH	G3	G4	G6	G3	G4	G6
Timed Race	Race Time (s)	−0.13	0.05	−0.62 **	−0.20	−0.89 **	−0.88 **	−0.97 **	−0.92 **	−0.53 *	−0.75 **	−0.70 **	0.62 **	0.28	0.83 **
PWT	TTE (s)	0.25	−0.14	0.43	−0.14	0.70 **	0.76 **	0.78 **	0.79 **	0.30	0.59 **	0.42	−0.64 **	−0.33	−0.77 **
	VO_2max_ (mL·min^−1^)	0.41	−0.34	0.35	0.05	0.79 **	0.75 **	0.73 **	0.76 **	0.58 **	0.58 **	0.53 *	−0.39	−0.36	−0.61 **
	VO_2max_/kg (mL·kg^−1^·min^−1^)	0.04	−0.02	0.28	−0.22	0.32	0.29	0.32	0.39	−0.06	0.05	0.01	−0.54 *	−0.48 *	−0.43
	%VO_2max_ at VT (%)	0.07	−0.05	0.20	0.36	0.59 **	0.51 *	0.57 *	0.43	0.54 *	0.48 *	0.44	−0.14	−0.05	−0.43
	%VO_2max_ at RCT (%)	0.17	−0.06	0.08	0.26	0.45	0.48 *	0.50 *	0.42	0.25	0.34	0.18	−0.33	−0.27	−0.55 *
DPT	TTE (s)	0.14	−0.07	0.50 *	0.00	0.81 **	0.81 **	0.85 **	0.85 **	0.47 *	0.64 **	0.54 *	−0.55 *	−0.34	−0.76 **
	VO_2peak_ (mL·min^−1^)	0.34	−0.29	0.46 *	−0.05	0.81 **	0.82 **	0.78 **	0.81 **	0.58 **	0.68 **	0.55 *	−0.42	−0.29	−0.66 **
	VO_2peak_/kg (mL·kg^−1^·min^−1^)	0.04	−0.02	0.52 *	−0.30	0.56 *	0.61 **	0.59 **	0.67 **	0.15	0.39	0.20	−0.62 **	−0.45	−0.66 **
	%VO_2peak_/VO_2max_ (%)	−0.03	0.04	0.38	−0.15	0.33	0.44	0.40	0.42	0.19	0.43	0.22	−0.25	−0.02	−0.38
SET	Mean power (W)	−0.06	0.03	0.54 *	0.03	0.51 *	0.47 *	0.53 *	0.59 **	0.40	0.29	0.48 *	−0.20	−0.33	−0.36
	Mean power/kg (W·kg^−1^)	−0.37	0.27	0.54 *	−0.07	0.19	0.19	0.27	0.34	0.08	0.00	0.18	−0.17	−0.36	−0.21

**Table 4:** The association between G3 technical characteristics and physiological parameters considering terrain. ** *p* < 0.01, * *p* < 0.05.

		Skiing Velocity (m/s)		Cycle Length (m)		Cycle Time (s)	
		Intermediate	Uphill	Intermediate	Uphill	Intermediate	Uphill
Timed Race	Race Time (s)	−0.92 **	−0.87 **	−0.57 *	−0.56 *	0.56 *	0.65 **
PWT	TTE (s)	0.72 **	0.68 **	0.31	0.35	−0.61 **	−0.62 **
	VO_2max_ (mL·min^−1^)	0.76 **	0.67 **	0.55 *	0.49 *	−0.33	−0.42
	VO_2max_/kg (mL·min^−1^·kg^−1^)	0.31	0.37	−0.08	−0.01	−0.53 *	−0.54 *
	%VO_2max_ at VT (%)	0.62 **	0.59 **	0.54 *	0.58 **	−0.14	−0.19
	%VO_2max_ at RCT (%)	0.45	0.44	0.22	0.22	−0.34	−0.37
DPT	TTE (s)	0.83 **	0.81 **	0.45	0.58 **	−0.56 *	−0.53 *
	VO_2peak_ (mL·min^−1^)	0.79 **	0.72 **	0.53 *	0.54 *	−0.40	−0.43
	VO_2peak_/kg (mL·min^−1^·kg^−1^)	0.56 *	0.62 **	0.10	0.29	−0.64 **	−0.58 **
	%VO_2peak_/VO_2max_ (%)	0.35	0.35	0.16	0.28	−0.29	−0.21
SET	Mean power (W)	0.52 *	0.55 *	0.43	0.54 *	−0.16	−0.19
	Mean power/kg (W·kg^−1^)	0.24	0.37	0.12	0.36	−0.17	−0.13

## Data Availability

The datasets generated and analyzed during the current study are available from the corresponding author upon reasonable request.

## Abbreviations

The following abbreviations are used in this manuscript:

FIS	International Ski Federation
DH	Downhill technique
DP	Double poling
DPT	Double pole test
GNSS	Global Navigation Satellite System
Mean power/kg	Relative mean power per body weight
PWT	Pole walk and run test
RCT	Respiratory compensation threshold
SD	Standard deviation
SET	Ski ergometer test
TTE-DPT	Time to exhaustion in the double pole test
TTE-PWT	Time to exhaustion in the pole walk and run test
VO_2max_	Absolute maximal oxygen uptake
VO_2max_/kg	Relative maximal oxygen uptake per body weight
VO_2peak_	Absolute peak oxygen uptake
VO_2peak_/kg	Relative peak oxygen uptake per body weight
VT	Ventilatory threshold
